# Factors Associated with Early ED Return Among Patients Discharged After Treatment for Alcohol Withdrawal Syndrome

**DOI:** 10.5811/westjem.63088

**Published:** 2026-10-11

**Authors:** Blake Briggs, Logan Smith, Ryan Green, Amirrez Nikbakht, David Cline

**Affiliations:** * University of Tennessee Health Science Center Knoxville, Division of Emergency Medicine, Knoxville, Tennessee; † Universty of Tennessee Health Science Center Memphis, College of Medicine, Memphis, Tennessee; ‡ Wake Forest University, School of Medicine, Winston-Salem, North Carolina

## Abstract

**Introduction:**

Alcohol withdrawal syndrome (AWS) is a frequent cause of emergency department (ED) visits, and some patients return soon after discharge. Identifying factors associated with short-term ED return may inform targeted interventions. Our primary objective was to identify factors associated with 72-hour ED return and 30-day hospital admission among patients discharged after treatment for AWS.

**Methods:**

We conducted a retrospective review of 318 ED encounters for AWS at a single academic medical center from January 1, 2020, to December 31, 2023. Demographic, psychiatric, housing, insurance, and discharge pharmacology variables were collected. We evaluated ED pharmacologic treatment strategy and discharge taper prescriptions. The primary outcome measure was 72-hour ED return, and the secondary outcome measure was hospital admission within 30 days. We compared groups using chi-square, Shapiro-Wilk, Fisher exact, and Mann–Whitney *U* tests. Variables with significant univariate associations were included in multivariable logistic regression, and results were reported as odds ratios (OR) with 95% confidence intervals.

**Results:**

Fourteen patients (4.4%) returned within 72 hours, and 32 patients (10.1%) were admitted within 30 days. On multivariable logistic regression, schizophrenia (OR, 8.59; 95% CI, 2.07–35.61), Medicaid insurance (OR, 4.43; 95% CI, 1.39–14.14), and anxiety (OR, 3.53; 95% CI, 1.10–11.39) were independently associated with increased 72-hour ED return. Homelessness was independently associated with higher 30-day admission (OR, 3.19; 95% CI, 1.26–8.07). Discharge tapers were not associated with either outcome.

**Conclusion:**

Among patients discharged after ED treatment for alcohol withdrawal syndrome, Medicaid insurance, anxiety, schizophrenia, and homelessness were associated with short-term return or admission. These findings suggest that social and psychiatric vulnerability may contribute to short-term ED reuse following discharge after AWS treatment. These results highlight opportunities to strengthen follow-up pathways and to address social determinants of health. Larger, multicenter studies are needed to clarify whether these associations are specific to AWS or reflect broader patterns of ED utilization.

## INTRODUCTION

Alcohol use disorder affects nearly 29% of the US population over a lifetime.^1^ Among individuals with chronic alcohol use, nearly half will experience alcohol withdrawal syndrome (AWS) upon cessation.^2^ Alcohol withdrawal syndrome substantially contributes to emergency department (ED) resource utilization; based on national estimates, it accounts for roughly .28% of all ED visits, translating to nearly 400,000 encounters annually in the US.^3^,^4^ This patient population often requires close monitoring and repeated medication administration during ED treatment. The cyclical nature of alcohol dependence and withdrawal symptoms further complicate care. On average, adults require more than five attempts to achieve sustained abstinence, underscoring the potential for repeated withdrawal episodes and recurrent ED presentations.^5^

While several studies have examined characteristics of frequent ED users, few have specifically focused on patients discharged after treatment for AWS. A systematic review by Giannouchos et al found that patients with alcohol dependence who have publicly provided insurance and comorbid psychiatric illness are disproportionately represented among frequent ED users.^6^ However, this analysis did not examine short-term return visits following AWS treatment in the ED. Patients discharged after acute alcohol withdrawal management are at high risk for recurrence, facing complex clinical and social barriers.^6^

Alcohol use disorder is disproportionately associated with psychiatric illness, unstable housing, and limited access to outpatient care, all of which are known contributors to recurrent ED utilization across multiple patient populations. Because alcohol use disorder is a chronic and relapsing condition, short-term ED return after AWS treatment may reflect expected disease trajectory, barriers to outpatient management, or unmet symptom control rather than a discrete “failure” of ED care. This knowledge gap is directly aligned with current research priorities identified by the Society for Academic Emergency Medicine (SAEM) GRACE initiative. This initiative highlighted the need to identify ED subgroups that may benefit from adjunctive or monotherapy phenobarbital use, evaluate postdischarge outcomes, and identify implementation barriers.^7^

Accordingly, in this retrospective cohort study, we evaluated factors associated with AWS-related return encounters to identify potentially high-risk subgroups for follow-up and linkage to care. This study represents a secondary analysis of a previously published retrospective cohort evaluating ED treatment strategies for AWS.^8^ We examined psychiatric comorbidity, housing status, insurance type, and the prescription of phenobarbital or benzodiazepines at discharge. We hypothesized that return visits would be more likely among patients with psychiatric illness, unstable housing, lack of insurance, or those who did not receive a prescription for a pharmacologic taper.

## METHODS

### Study Design and Setting

This study represents a secondary analysis of a previously published retrospective cohort evaluating ED outcomes among patients treated for AWS with phenobarbital or benzodiazepines.^8^ The original study examined admission rates, ED length of stay, and treatment strategies across all AWS encounters. The current analysis focuses specifically on the subset of patients who were discharged from the ED after receiving intravenous (IV) pharmacologic treatment for AWS, with the objective of identifying factors associated with early ED return and subsequent hospital admission. We conducted the study at a single regional academic medical center and tertiary care referral center with an annual ED volume of approximately 79,000 visits. The institutional review board reviewed the protocol and deemed it exempt from full review due to minimal risk and use of deidentified data.

**Population Health Research Capsule**

What do we already know about this issue?

*Patients discharged after emergency department (ED) alcohol withdrawal treatment may return early, but factors associated with short-term return are poorly defined.*

What was the research question?

*What factors are associated with 72-hour ED return after discharge for alcohol withdrawal syndrome?*

What was the major finding of the study?

*Schizophrenia had the highest odds of 72-hour return (OR, 8.59; 95% CI, 2.07*–*35.61;* P *= .003) and homelessness was independently associated with 30-day admission (OR, 3.19; 95% CI, 1.26*–*8.07).*

How does this improve population health?

*Identifying psychiatric and social risk factors may help target follow-up and reduce repeat ED use after alcohol withdrawal care.*

### Selection of Participants and Interventions

We identified adult patients at least 18 years of age who presented to the ED between January 1, 2020, and December 31, 2023, using a structured query of the electronic health record (EHR). We identified cases through an administrative flag triggered by ordering the “ED alcohol withdrawal” order set. We selected this timeframe as a convenience sample to allow for seasonal variation and to reflect the first full year phenobarbital became available at our institution for AWS treatment. Inclusion required activation of the institutional AWS treatment pathway during the index ED encounter, although patients may have presented with concurrent medical complaints. For analysis, patients were categorized into three mutually exclusive exposure groups: phenobarbital-only (received IV phenobarbital but no benzodiazepines during the ED visit); benzodiazepine-only (received IV benzodiazepine but no phenobarbital); or combination therapy (received IV phenobarbital plus benzodiazepine during the same encounter). Encounters were categorized into phenobarbital-only, benzodiazepine-only, or combination therapy groups based on medications administered during the index ED visit. Benzodiazepine administration was determined by the treating clinician and was not protocolized to a fixed cumulative dose.

Phenobarbital dosing followed the institutional treatment pathway described above. The decision to administer phenobarbital, benzodiazepines, or combination therapy was at the discretion of the treating emergency physician. Phenobarbital dosing at our institution followed a standardized pathway consisting of an initial 260 mg intravenous (IV) infusion administered over 30 minutes, with an optional additional 130 mg IV dose administered 30 minutes later at clinician discretion. The following benzodiazepines were available in our ED: lorazepam (IV, intramuscular (IM)); diazepam (IV, IM); and midazolam (IV). These were administered according to clinician judgment and typical practice. Any dose of a given medication from the “ED alcohol withdrawal” order set was considered an exposure for the purpose of analysis.

Inclusion criteria were as follows: receipt of IV phenobarbital; IV benzodiazepine; or both in the ED; and treatment for AWS with subsequent discharge from the ED. Admission decisions were made at clinician discretion based on overall clinical severity, symptom progression, comorbidities, social factors, and anticipated outpatient management needs, consistent with routine ED practice. Exclusion criteria included the following: patients younger than 18 years of age; encounters managed primarily by the trauma surgery service following trauma activation; cases in which patients were not treated for AWS despite order set use, patients intubated prior to receiving any AWS-directed medications; and those admitted to the hospital. Patients treated exclusively with oral regimens were excluded because these encounters generally represented lower-acuity AWS presentations and had less standardized documentation of symptom severity and treatment administration. Gabapentin-based withdrawal protocols were not used within the institutional AWS pathway during the study period and were therefore not analyzed separately.

In cases where patients were discharged with a prescription to manage withdrawal symptoms, these were administered according to clinician judgment and typical practice. Specifically, phenobarbital tapers were typically four-day courses: Day 1, 60 mg four times daily (QID); Day 2, 60 mg three times daily (TID); Day 3, 60 mg twice daily (BID); and Day 4, 30 mg BID. In some cases, a fifth day of 30 mg BID was added at the clinician’s discretion. Benzodiazepine prescriptions were most commonly for chlordiazepoxide, with variable dosing regimens. Some patients received as-needed dosing of 25–50 mg every 4–8 hours for approximately 3–5 days. Others received a scheduled taper, typically: Day 1, 50 mg TID; Days 2–3, 25 mg BID; and Day 4, a single 25 mg dose.

Insurance status was determined from patient intake forms completed at registration. For simplicity and to allow for structured data analysis, insurance type was grouped into the following classifications: private, Medicare, Medicaid, and uninsured. Before data abstraction, we consulted the hospital’s registration and billing departments to ensure consistent grouping of insurance carriers into these predefined classifications. Housing status was assessed from the same intake documentation and verified with case management or social work notes when available. Psychiatric comorbidity was identified using either a previously documented diagnosis in the EHR or the presence of a *corresponding International Classification of Diseases*, 10^th^ Rev, diagnosis code during the index encounter. Prior to data abstraction, the analysis focused on commonly documented psychiatric conditions including anxiety, depression, and schizophrenia-spectrum disorders to improve consistency of retrospective abstraction within this cohort.

The alcohol withdrawal order set was fully implemented in 784 ED encounters during the study period. Of these, seven of the 784 visits were excluded as the patient left against medical advice prior to a determination of safe discharge or admission by the treating emergency physician. Of the remaining 777 visits, 459 were admitted. Therefore, our final study cohort included 318 patient encounters for alcohol withdrawal management who were discharged from the ED (Figure 1). This study cohort represents a selected subgroup of AWS encounters discharged after IV treatment and does not reflect the total number of alcohol-related ED presentations within the health system during the study period.

### Measurements

Strengthening the Reporting of Observational Studies in Epidemiology guidelines were followed to ensure adequate data collection and analysis.^9^ This chart review followed recommended methodological standards for retrospective medical record review studies as described by Worster and Bledsoe, including use of standardized data abstraction forms, trained abstractors, and predefined variable definitions. We developed and pilot-tested a standardized data abstraction form, along with a study manual detailing explicit variable definitions. Variables for this analysis included demographics, active and historical mental health conditions, insurance status, housing status, medication exposures, disposition, and return visits. We selected variables based on prior literature evaluating return ED visits and clinically relevant factors reliably identifiable through retrospective chart review.

Abstractor training and adjudication procedures followed published recommendations for medical record review.^11^ We followed broadly accepted literature in emergency medicine regarding defining variables, structured abstraction forms, and blinded reabstraction to reduce bias, enhance validity and interobserver reliability.^12^,^13^ Abstractors completed human subjects research training and one-on-one orientation sessions with the primary study investigator (BB) prior to beginning abstraction. Initial chart reviews were directly observed, with ongoing meetings to address uncertainties. To reduce bias, a senior abstractor independently rereviewed charts for key variables, with discrepancies adjudicated by the primary investigator. Cases with unresolved uncertainty or missing key data after adjudication were excluded. No imputation was performed.

### Outcomes

The primary outcomes were (1) return to the ED within 72 hours for AWS and (2) hospital admission within 30 days for AWS following the index ED visit. The 72-hour ED return time was selected to evaluate short-term recurrence following acute withdrawal treatment and represents a commonly used timeframe in emergency medicine outcomes research, while 30-day admission was selected to assess broader postdischarge healthcare utilization and clinical deterioration.

A return encounter was defined as any subsequent ED visit at our institution or a participating regional facility following the index visit. Return visits were further categorized as occurring within 0–3 days or an admission within 30 days after the index visit. We limited return encounters and admissions to visits related to AWS. To capture out-of-network returns, abstractors queried the East Tennessee Health Information Network, a regional health information exchange that provides access to encounter data from hospitals and clinics within a 60-mile radius.^14^

### Statistical Analysis

We summarized categorical variables as frequencies and percentages. For categorical comparisons, chi-square or Fisher exact tests were used, as appropriate, and unadjusted odds ratios (OR0 with 95% CI were calculated for significant associations. For continuous variables, independent-samples t-tests were used for normally distributed data and Mann–Whitney *U* tests for nonnormally distributed data. A two-sided α value of .05 assumed statistical significance, and all analyses were performed using SPSS, version 31 (IBM Corp). Because our primary aim was to determine which variables were independently associated with short-term return, only factors associated with significant univariate associations were included in the multivariable model to avoid overfitting. We constructed separate logistic regression models for 72-hour ED return and 30-day hospital admission.

## RESULTS

### Demographics and Clinical Characteristics

A total of 318 patients met inclusion criteria, with a 72-hour ED return rate for alcohol withdrawal of 14 patients (4.4%) and a 30-day admission rate for 32 patients (10.1%). The 72-hour ED return rate was 3.3% in women (2/61) and 4.7% in men (12/257; *P* = 1.00). The 30-day admission rate was 8.2% in women (5/61) and 10.5% in men (27/257; χ^2^ (1, *n* = 318) = .29, *P* = .59). Across race groups, the 72-hour ED return rate was 5.3% in Black patients (1/19), 0% in Latino (0/2) and Native American patients (0/1), and 4.4% in White patients (13/296; χ^2^ (3, *n* = 318) = 0.30, *P* = .96). The corresponding 30-day admission proportions for these groups were 21.1% (4/19), 0% (0/2), 0% (0/1), and 9.5% (28/296), respectively (χ^2^ (3, *n* = 318) = 2.75, *P* = .43).

### Mental Health Conditions

Patients with anxiety had a significantly higher 72-hour return rate (8.9%, 7/79) compared to those without anxiety (2.9%, 7/239; χ^2^ (1, *n* = 318) = 4.96, *P* = .03; OR = 3.22, 95% CI, 1.09–9.49), but no difference in 30-day admission (11.4%, 9/79 versus 9.6%, 23/239, *P* = .65). Schizophrenia was also associated with increased 72-hour return (22.2%, 4/18 versus 3.3%, 10/300; *P* = .005; OR = 8.29, 95% CI, 2.31–29.3) without a difference in 30-day admission (16.7%, 3/18 versus 9.7%, 29/300, *P* = .41). Depression was not significantly associated with either outcome, with *P* = .27 for 72-hour return and *P* = .06 for 30-day admission. Similarly, mood disorder was not significant for 72-hour return (*P* = .38) or 30-day admission (*P* = 1.00). Posttraumatic stress disorder showed no significant association, with *P* = .08 for 72-hour return and *P* = .28 for 30-day admission. Neither was suicidal ideation significant for either outcome (*P* = 1.00 for 72-hour return; *P* = .56 for 30-day admission).

### Insurance and Housing

Medicaid coverage was associated with a higher 72-hour return rate (11.9%, 7/59 versus 2.7%, 7/259; χ^2^ (1, *n* = 318) = 9.58, *P* = .002; OR, 4.85; 95% CI, 1.63–14.40), but not 30-day admission (10.2%, 6/59 versus 10.0%, 26/259, *P* = .98). Housing status was significantly associated with 30-day admission, with homeless patients having a 25.0% (8/32) admission proportion versus an 8.4% (24/286) proportion for housed patients (χ^2^ (1, *n* = 318) = 8.77, *P* = .003; OR, 3.64; 95% CI, 1.48–8.98). We found no difference in 72-hour return between these groups (3.1%, 1/32 versus 4.5%, 13/286, *P* = 1.00). Private insurance was not significantly associated with either outcome, with *P* = 1.00 for 72-hour return and *P* = .30 for 30-day admission. Similarly, Medicare was not significant for 72-hour return (*P* = 1.00) or 30-day admission (*P* = .56). Uninsured status showed no significant association, with *P* = .07 for 72-hour return and *P* = .30 for 30-day admission.

### Benzodiazepine and Intravenous Phenobarbital Use

When encounters were grouped by treatment strategy (phenobarbital-only, benzodiazepine-only, or combination therapy), there were no significant associations with either 72-hour ED return (4.0%, 1.8%, and 10.5%, respectively; χ^2^ = 3.72, *P* = .16) or 30-day admission (8.9%, 12.5%, and 13.2%, respectively; χ^2^ = 1.04, *P* = .59). These findings were consistent with the results of our previously published cohort study, which showed similar return rates across treatment groups.

### Multivariable Analysis

We used multivariate regression analysis to determine whether potential confounding factors explained the association of our measured variables with admission. Table 1 presents the multivariable logistic regression results evaluating factors associated with 72-hour ED return among patients treated for AWS. Variables entered into the model were those with significant univariate associations. Schizophrenia (OR, 8.59; 95% CI, 2.07–35.61), Medicaid insurance (OR, 4.43; 95% CI, 1.39–14.14), and anxiety (OR, 3.53; 95% CI, 1.10–11.39) remained independently associated with 72-hour ED return.

Table 2 summarizes the multivariable logistic regression examining factors associated with 30-day hospital admission following ED discharge for AWS. Factors with significant association with admission on univariate analysis (or near significant association in the case of depression, *P* = .06) were entered into the model. Homelessness remained independently associated with 30-day admission (OR, 3.19; 95% CI, 1.26–8.07), whereas depression did not reach statistical significance.

Figure 2 summarizes the adjusted ORs and 95% CIs from the multivariable logistic regression analyses for factors associated with 72-hour ED return and 30-day hospital admission following ED discharge for AWS.

## DISCUSSION

In this single-center cohort of 318 patients discharged after treatment for AWS, we identified social and psychiatric factors, not pharmacologic strategy, as the primary factors associated with early ED return and hospital admission. Patients with Medicaid insurance were more likely to return to the ED within 72 hours, consistent with prior studies demonstrating that publicly insured patients face greater barriers to timely outpatient addiction care.^6^,^15^ Low socioeconomic status and limited access to longitudinal treatment likely contribute to this pattern.^16^ Uninsured patients did not show higher short-term return rates, a finding that may reflect financial deterrents to ED utilization rather than improved access to care.

Prior literature examining short-term outcomes after ED treatment of AWS is limited. One of the largest cohorts to date is the study by Lebin et al, which evaluated approximately 470 ED patients treated for AWS and examined return encounters primarily in relation to treatment strategy (phenobarbital versus benzodiazepines).^17^ In comparison, our study of 318 discharged encounters represents one of the largest cohorts evaluating factors associated with early ED return and 30-day hospital admission following AWS treatment. Unlike prior studies that focuses primarily on pharmacologic management, our analysis examined patient-level vulnerability, including psychiatric comorbidity and social determinants of health. Other literature addressing ED revisits after alcohol-related presentations has emphasized the importance of withdrawal severity, mental illness, unstable housing, and barriers to follow-up care, supporting the associations observed in this cohort.^3^,^18^ These prior studies are limited in scope, with some being narrative reviews or commentaries on revisits, rather than a true retrospective, single-cohort investigation.

Our findings also align with the work of Scheuermeyer et al, who reported that patients presenting to the ED with alcohol withdrawal or alcohol-related conditions frequently return for subsequent care and often face substantial psychiatric and social vulnerability.^19^ However, unlike the broader alcohol-related ED cohort described by Scheuermeyer et al, our study focused specifically on patients discharged after treatment for AWS and evaluated AWS-related return encounters and subsequent hospital admission. The 72-hour ED return rate in our cohort (4.4%) was lower than the 15% one-week revisit rate reported by Scheuermeyer et al.

Several factors may explain this difference. First, our study evaluated a narrower 72-hour outcome window, whereas Scheuermeyer et al examined revisits occurring within seven days of discharge. Additionally, our cohort was limited to patients discharged after IV treatment for AWS and evaluated AWS-related return encounters specifically, while the Scheuermeyer cohort included a broader population of alcohol withdrawal and alcohol-related ED presentations. Differences in healthcare systems, institutional treatment pathways, and unmeasured withdrawal severity may have also contributed to the differing revisit rates.

Homelessness was not associated with early ED return but was linked to increased 30-day hospital admission. Our findings suggest that acute relapse or social instability often escalates to inpatient care over a longer horizon. These findings suggest that housing instability may be associated with increased healthcare utilization following ED discharge for AWS. Among psychiatric comorbidities, anxiety and schizophrenia were independently associated with early return, aligning with previous literature reporting that frequent ED users have a disproportionate burden of psychiatric illness.^6^ The absence of a parallel increase in 30-day admissions suggests an opportunity for improved outpatient psychiatric linkage and postdischarge support to reduce short-term ED recidivism.

These associations remained significant in multivariable logistic regression, indicating that Medicaid insurance, schizophrenia, and anxiety were independently associated with 72-hour return after adjusting for other factors. Likewise, homelessness was independently associated with 30-day admission. The persistence of these associations after adjustment suggests that these social and psychiatric variables exert effects that are not explained by age, sex, or comorbidity distribution but instead reflect true underlying risk factors for short-term recidivism. These associations may reflect broader patterns of ED utilization and structural vulnerability rather than mechanisms unique to alcohol withdrawal syndrome alone.

Neither discharge prescription tapers, nor ED IV medication strategy significantly affected return or admission outcomes. When categorized by phenobarbital-only, benzodiazepine-only, or combination therapy, no differences were observed in 72-hour ED return or 30-day readmission rates. These findings mirror our previously published phenobarbital outcomes study and reinforce that social and psychiatric determinants, rather than pharmacologic regimens, showed stronger associations with short-term outcomes in this patient cohort. While medication choice is a critical component of acute stabilization, postdischarge outcomes in these patients appear to depend more on systemic factors such as access to follow-up care, psychiatric comorbidity, and housing stability. Per institutional practice, patients presenting to the ED with alcohol use disorder or AWS were routinely provided discharge resources including contact information for outpatient addiction clinics, inpatient rehabilitation programs, and community detoxification services. During weekday daytime hours, ED social workers were available to assist with disposition planning, facilitate referral to inpatient or outpatient treatment programs, and provide additional substance use resources when appropriate.

To our knowledge, this is the first study to specifically examine short-term outcomes in patients with AWS discharged from the ED. Our results expand the evidence for ED management of AWS by identifying high-risk subgroups. These high-risk patients—including those who have been diagnosed with psychiatric illness, are covered by Medicaid insurance, and are experiencing homelessness—may benefit from enhanced discharge planning and coordinated addiction or social services. These findings indicate that social and psychiatric factors demonstrated stronger associations with return encounters than ED pharmacologic treatment strategy. Effective interventions could come more from improved continuity of care, access to addiction resources, and stabilization of social determinants.

These findings directly align with the key priorities outlined by the SAEM GRACE initiative, which has called for rigorous, patient-centered research in AWS.^7^ These priorities include identifying patient subgroups most likely to benefit from adjunctive or monotherapy phenobarbital, understanding postdischarge outcomes, and assessing barriers to implementation. Our findings suggest that recurrence risk after ED discharge is driven primarily by social determinants and comorbidity rather than treatment regimen, which supports the GRACE agenda and underscores the need for interventions beyond pharmacologic management to improve outcomes in this population. Future research should prioritize linkage to outpatient addiction and social services for outcome measurements, as well as multicenter analysis for further validation of this study’s findings. Additional studies are needed to better evaluate the role of outpatient pharmacologic interventions.

## LIMITATIONS

This study has several limitations. It was conducted at a single academic center, which may limit generalizability to other settings. The relatively small number of outcome events limited model complexity and may reduce external validity and precision of estimated associations. The retrospective design introduces potential confounding and selection bias, since physician preference likely influenced who received a discharge taper and which regimen was chosen. Benzodiazepine selection and cumulative dosing varied between encounters and were not standardized or converted into dose-equivalent comparisons, which may have limited interpretation of treatment strategy analyses. There was heterogeneity among the taper prescriptions, including variations in dosage and duration. Medication adherence postdischarge was not measured, and the sample sizes were small, limiting statistical power. Structured outpatient follow-up data were unavailable, constraining assessment of long-term outcomes beyond return visits captured in the regional health information network.

This study evaluated psychiatric comorbidities documented at discharge; however, the severity of the underlying psychiatric illness was not captured. Additional psychiatric conditions, including schizoaffective disorder and bipolar disorder with psychotic features, were not separately analyzed, which may limit characterization of psychiatric comorbidity within the cohort. Social determinants such as housing instability may also have influenced clinician disposition decisions, potentially affecting which patients were discharged versus admission. Finally, although we used the East Tennessee Health Information Network to capture return visits across regional hospitals, some return encounters may have occurred outside the network and gone unrecorded. Collectively, these limitations mean our findings should be viewed as exploratory rather than definitive.

## CONCLUSION

Among patients discharged from the ED after treatment for alcohol withdrawal syndrome, variables such as Medicaid insurance, anxiety, and schizophrenia-spectrum disorders were associated with return within 72 hours. Homelessness was associated with a higher rate of 30-day hospital admission. Pharmacologic discharge tapers were not associated with differences in either short-term ED return or 30-day admission. These findings should be interpreted within the limitations of a single-center retrospective study but suggest that social and psychiatric factors may play a greater role in postdischarge outcomes than medication strategy alone. Future multicenter work is needed to confirm these associations and to explore interventions that strengthen outpatient follow-up and address social determinants of health in this high-risk population.

## Figures and Tables

**Figure 1:**
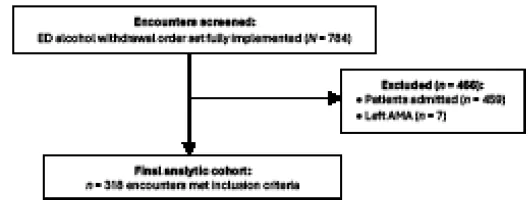
Flow diagram of patient selection in a retrospective cohort study evaluating factors associated with 72-hour emergency department return and 30-day hospital admission among patients treated for alcohol withdrawal syndrome. *AMA*, against medical advice; *ED*, emergency department.

**Figure 2:**
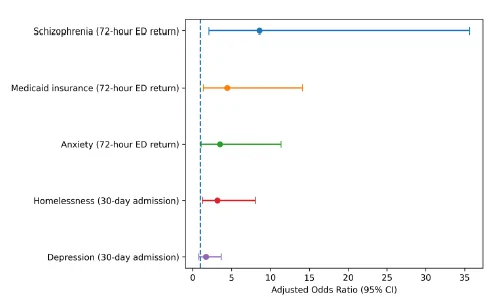
Adjusted odds ratios for factors associated with 72-hour emergency department return and 30-day hospital admission after treatment for alcohol withdrawal syndrome. *ED*, emergency department.

**Table 1:** Multivariable logistic regression analysis of factors associated with 72-hour emergency department return in a retrospective cohort of patients treated for alcohol withdrawal syndrome.

Parameter	Odds Ratio	95% CI	*P* value
Schizophrenia	8.59	2.07–35.61	.003*
Medicaid insurance	4.43	1.39–14.14	.01*
Anxiety	3.53	1.10–11.39	.03*

* Statistically significant results (*P* < .05).

**Table 2:** Multivariable logistic regression analysis of factors associated with 30-day hospital admission in a retrospective cohort of patients treated for alcohol withdrawal syndrome.

Parameter	Odds Ratio	95% CI	*P* value
Homelessness	3.18	1.26–8.07	.01*
Patient depression	1.70	0.79–2.69	.18

* Statistically significant results (*P* < .05).

## References

[1] Kranzler HR (2023). Overview of alcohol use disorder. Am J Psychiatry.

[2] Wood E, Albarqouni L, Tkachuk S (2018). Will this hospitalized patient develop severe alcohol withdrawal syndrome? The rational clinical examination systematic review. JAMA.

[3] Gottlieb M, Chien N, Moyer E (2025). Alcohol withdrawal syndrome presentations to emergency departments in the United States from 2015 to 2023. Acad Emerg Med.

[4] Cairns C, Kang K (2022). National Hospital Ambulatory Medical Care Survey: 2022 Emergency Department Summary Tables.

[5] Kelly JF, Greene MC, Bergman BG (2019). How many recovery attempts does it take to successfully resolve an alcohol or drug problem? Estimates and correlates from a national study of recovering US adults. Alcohol Clin Exp Res.

[6] Giannouchos TV, Kum HC, Foster MJ (2019). Characteristics and predictors of adult frequent emergency department users in the United States: A systematic literature review. J Eval Clin Pract.

[7] Punia K, Scott W, Manuja K (2024). SAEM GRACE: Phenobarbital for alcohol withdrawal management in the emergency department: a systematic review of direct evidence. Acad Emerg Med.

[8] Briggs B, Smith L, Yates N (2026). Fewer admissions, shorter stays: phenobarbital use for alcohol withdrawal in the emergency department. Acad Emerg Med.

[9] von Elm E, Altman DG, Egger M (2008). Strengthening the reporting of observational studies in epidemiology (STROBE) statement: Guidelines for reporting observational studies. J Clin Epidemiol.

[10] Worster A, Bledsoe RD, Cleve P (2005). Reassessing the methods of medical record review studies in emergency medicine research. Ann Emerg Med.

[11] Worster A, Haines T (2004). Advanced statistics: understanding medical record review (MRR) studies. Acad Emerg Med.

[12] Gilbert EH, Lowenstein SR, Koziol-McLain J (1996). Chart reviews in emergency medicine research: Where are the methods?. Ann Emerg Med.

[13] Kaji AH, Schriger D, Green S (2014). Looking through the retrospectoscope: Reducing bias in emergency medicine chart review studies. Ann Emerg Med.

[14] East Tennessee Health Information Network What We Do.

[15] DuBard CA, Yow A, Bostrom S (2009). Racial/ethnic differences in quality of care for North Carolina Medicaid recipients. N C Med J.

[16] McMaughan DJ, Oloruntoba O, Smith ML (2020). Socioeconomic status and access to healthcare: interrelated drivers for healthy aging. Front Public Health.

[17] Lebin JA, Mudan A, Murphy CE (2022). Return encounters in emergency department patients treated with phenobarbital versus benzodiazepines for alcohol withdrawal. J Med Toxicol.

[18] Wolf C, Curry A, Nacht J (2020). Management of alcohol withdrawal in the emergency department: current perspectives. Open Access Emerg Med.

[19] Scheuermeyer FX, Lane D, Grunau B (2023). Risk factors associated with 1-week revisit among emergency department patients with alcohol withdrawal. CJEM.

